# Scm^6^A: A Fast and Low-cost Method for Quantifying m^6^A Modifications at the Single-cell Level

**DOI:** 10.1093/gpbjnl/qzae039

**Published:** 2024-06-07

**Authors:** Yueqi Li, Jingyi Li, Wenxing Li, Shuaiyi Liang, Wudi Wei, Jiemei Chu, Jingzhen Lai, Yao Lin, Hubin Chen, Jinming Su, Xiaopeng Hu, Gang Wang, Jun Meng, Junjun Jiang, Li Ye, Sanqi An

**Affiliations:** Department of Biochemistry and Molecular Biology, School of Basic Medicine Sciences, Guangxi Medical University, Nanning 530021, China; Key Laboratory of Biological Molecular Medicine Research, Education Department of Guangxi Zhuang Autonomous Region, Nanning 530021, China; Department of Pathology, Guangdong Second Provincial General Hospital, Guangzhou 510317, China; Life Sciences Institute & Guangxi Key Laboratory of AIDS Prevention and Treatment, Guangxi Medical University, Nanning 530021, China; Department of Systems Biology, Columbia University Medical Center, New York, NY 10032, USA; Department of Bioinformatics, Anjin Biotechnology Co., Ltd., Guangzhou 510000, China; Life Sciences Institute & Joint Laboratory for Emerging Infectious Diseases in China (Guangxi)-ASEAN, Guangxi Medical University, Nanning 530021, China; Life Sciences Institute & Guangxi Key Laboratory of AIDS Prevention and Treatment, Guangxi Medical University, Nanning 530021, China; Life Sciences Institute & Guangxi Key Laboratory of AIDS Prevention and Treatment, Guangxi Medical University, Nanning 530021, China; Life Sciences Institute & Guangxi Key Laboratory of AIDS Prevention and Treatment, Guangxi Medical University, Nanning 530021, China; Life Sciences Institute & Guangxi Key Laboratory of AIDS Prevention and Treatment, Guangxi Medical University, Nanning 530021, China; Life Sciences Institute & Guangxi Key Laboratory of AIDS Prevention and Treatment, Guangxi Medical University, Nanning 530021, China; Life Sciences Institute & Guangxi Key Laboratory of AIDS Prevention and Treatment, Guangxi Medical University, Nanning 530021, China; Life Sciences Institute & Guangxi Key Laboratory of AIDS Prevention and Treatment, Guangxi Medical University, Nanning 530021, China; Life Sciences Institute & Guangxi Key Laboratory of AIDS Prevention and Treatment, Guangxi Medical University, Nanning 530021, China; Life Sciences Institute & Guangxi Key Laboratory of AIDS Prevention and Treatment, Guangxi Medical University, Nanning 530021, China; Life Sciences Institute & Guangxi Key Laboratory of AIDS Prevention and Treatment, Guangxi Medical University, Nanning 530021, China; Department of Biochemistry and Molecular Biology, School of Basic Medicine Sciences, Guangxi Medical University, Nanning 530021, China; Key Laboratory of Biological Molecular Medicine Research, Education Department of Guangxi Zhuang Autonomous Region, Nanning 530021, China; Life Sciences Institute & Guangxi Key Laboratory of AIDS Prevention and Treatment, Guangxi Medical University, Nanning 530021, China

**Keywords:** Single-cell, *N* ^6^-methyladenosine, Heterogeneity, T cell, Machine learning

## Abstract

It is widely accepted that *N*^6^-methyladenosine (m^6^A) exhibits significant intercellular specificity, which poses challenges for its detection using existing m^6^A quantitative methods. In this study, we introduced Single-cell m^6^A Analysis (Scm^6^A), a machine learning-based approach for single-cell m^6^A quantification. Scm^6^A leverages input features derived from the expression levels of m^6^A *trans* regulators and *cis* sequence features, and offers remarkable prediction efficiency and reliability. To further validate the robustness and precision of Scm^6^A, we first applied Scm^6^A to single-cell RNA sequencing (scRNA-seq) data from peripheral blood mononuclear cells (PBMCs) and calculated the m^6^A levels in CD4^+^ and CD8^+^ T cells. We also applied a winscore-based m^6^A calculation method to conduct *N*^6^-methyladenosine sequencing (m^6^A-seq) analysis on CD4^+^ and CD8^+^ T cells isolated through magnetic-activated cell sorting (MACS) from the same samples. Notably, the m^6^A levels calculated by Scm^6^A exhibited a significant positive correlation with those quantified through m^6^A-seq in different cells isolated by MACS, providing compelling evidence for Scm^6^A’s reliability. Additionally, we performed single-cell-level m^6^A analysis on lung cancer tissues as well as blood samples from patients with coronavirus disease 2019 (COVID-19), and demonstrated the landscape and regulatory mechanisms of m^6^A in different T cell subtypes from these diseases. In summary, Scm^6^A is a novel, dependable, and accurate method for single-cell m^6^A detection and has broad applications in the realm of m^6^A-related research.

## Introduction

As the most widespread epigenetic modification in messenger RNA (mRNA), *N*^6^-methyladenosine (m^6^A) plays pivotal roles in gene expression regulation and is intricately linked to physiological processes in various diseases [1–7]. Among its multifaceted regulatory functions, m^6^A governs T cell differentiation and influences immune-related gene expression, garnering substantial attention [8]. To gain deeper insights into the roles of m^6^A in biological progresses, it becomes imperative to discern transcriptome-wide m^6^A levels and sites within individual cells. For instance, the presence of a multitude of immune cell and T cell subtypes [8–11], poses a formidable challenge, as current m^6^A detection methods designed for bulk cell populations fall short in characterizing m^6^A levels and sites at the single-cell level.

It is well-established that cell type-specific m^6^A levels and *de novo* m^6^A deposition are jointly regulated by *trans*-acting regulators and *cis*-regulatory elements [12]. In theory, leveraging information on these *trans*-acting regulators and *cis*-regulatory elements as input enables the prediction of m^6^A at the single-cell level using computational methods. Machine learning and other computational approaches have found extensive application in the analysis of diverse omics data, significantly advancing our understanding of biology [13–17]. In theory, machine learning holds the promise of predicting RNA methylation level at the single-cell level. In our prior research, we developed a computational framework to systematically identify *trans* regulators of m^6^A and performed experiments to verify the reliability of these *trans* regulators [18]. Additionally, a reliable regulatory network from *trans* regulators to m^6^A sites was constructed. Furthermore, we identified cell-specific m^6^A *cis*-regulatory motifs [18]. Machine learning, as a potent predictive tool, has been extensively employed in forecasting gene expression, DNA methylation, and alternative splicing, leveraging multiple biological features with impressive accuracy [19–22]. In fact, Xue et al. highlighted the challenges tied to the experimental detection of RNA m^6^A. To address this, they investigated the possibility of using computational methods to predict RNA methylation status based on gene expression data. By employing methods such as support vector machine (SVM) and random forest (RF), Xue et al. determined that gene expression data can indeed act as a reliable predictor for m^6^A methylation status [23]. Their findings have convinced us of the viability of predicting single-cell m^6^A using machine learning methods grounded in gene expression level. Herein, we attempted to develop a single-cell-level m^6^A calculation method through a machine learning method.

In this study, we leveraged comprehensive information on the *trans* regulators and *cis* elements (including motif and sequence data) of m^6^A to create a machine learning-based quantitative method for single-cell m^6^A analysis, named Single-cell m^6^A Analysis (Scm^6^A, https://github.com/Ansanqi/Scm6A). We applied multiple machine learning methods to establish the associations of *trans* regulators and *cis* sequence features with m^6^A levels in single cells. Subsequently, Scm^6^A was established with substantial predictive power to predict the level of m^6^A in individual cells. After that, we applied Scm^6^A to single-cell RNA sequencing (scRNA-seq) data from peripheral blood mononuclear cells (PBMCs) and calculated the m^6^A levels in CD4^+^ and CD8^+^ T cells. To validate the accuracy and reliability of Scm^6^A, we also performed *N*^6^-methyladenosine sequencing (m^6^A-seq) on CD4^+^ and CD8^+^ T cells, isolated via magnetic-activated cell sorting (MACS), from the same donors. Our findings underscored the precision and dependability of Scm^6^A in discerning single-cell m^6^A levels, in comparison to m^6^A-seq results derived from MACS-isolated cell populations. Subsequently, we extended our analysis to investigate single-cell m^6^A profiles in lung cancer scRNA-seq data using Scm^6^A and demonstrated that the m^6^A profiles are highly heterogeneous at the single-cell level in different subtypes of T cells in lung cancer. We also applied our model to single-cell dataset of the coronavirus disease 2019 (COVID-19) [24], and demonstrated its good performance in classifying T cells and B cells. Furthermore, we compared our Scm^6^A with the experimental method single-cell *N*^6^-methyladenosine sequencing (scm^6^A-seq) developed by the Yang et al. [25], and found that Scm^6^A not only performed well in mouse cells but also exhibited a significant correlation with experimental sequencing results.

## Results

### RF outperformed other machine learning models in single-cell m^6^A calculation

**Figure 1:**
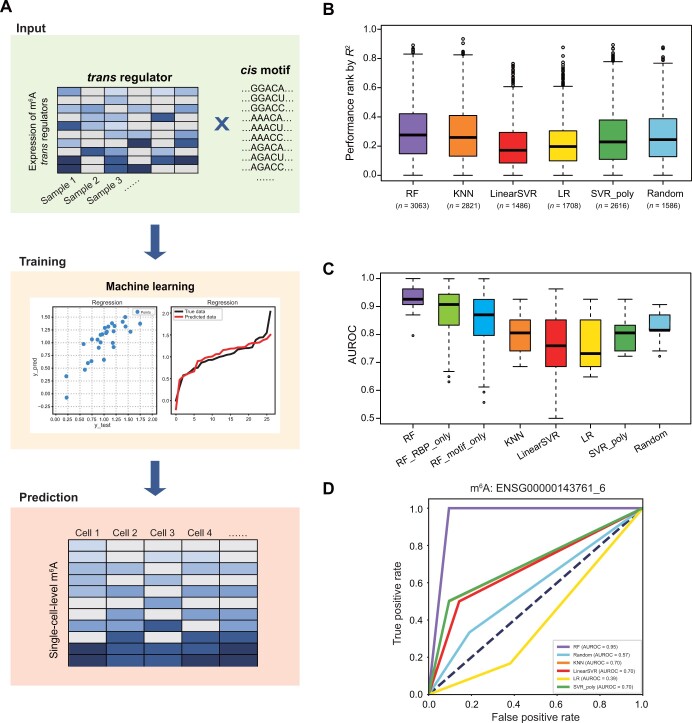
Evaluation of single-cell m^6^A prediction models **A**. Algorithmic framework for single-cell m^6^A calculation. **B**. Box plot showing the performances of different models ranked by *R*^2^ in the test set. **C**. Box plot showing the AUROC values for different models. **D**. ROC curves and AUROC values for different models in prediction of m^6^A sites in EMSG00000143761-6. AUROC, area under the receiver operating characteristic curve; ROC, receiver operating characteristic; KNN, K-nearest neighbor; SVR_poly, support vector regression with the poly kernel; LR, linear regression; LinearSVR, linear support vector regression; RF, random forest; m^6^A, *N*^6^-methyladenosine; RBP, RNA-binding protein.

**Figure 1**A illustrates the workflow of our study. Initially, we first collected the gene expression data of 593 reliable m^6^A regulators as well as the conserved sequence features associated with m^6^A which we validated before [18]. To establish a precise single-cell m^6^A calculation model, we evaluated five machine learning regression models, including RF, K-nearest neighbor (KNN), support vector regression (SVR) with the poly kernel (SVR-poly), linear regression (LR), and linear SVR (LinearSVR). All optimal parameters of the abovementioned models were obtained by grid search based on the *trans* and *cis* data (Table S1).

The coefficient of determination (*R*^2^), commonly employed to gauge the performance of regression-based machine learning models, was utilized as an evaluative parameter for assessing the proximity of data points to the fitted line. Notably, our analysis showed that the RF model consistently outperformed the other machine learning models, displaying higher *R*^2^ values (Figure 1B), indicating that the RF model is the most suitable for single-cell m^6^A prediction. Additionally, the correlation analysis between the predicted m^6^A levels and true m^6^A levels demonstrated the superior reliability of the RF model-based m^6^A calculation method compared to other models (Figure S1A). By defining the difference between the predicted value and the actual value as binary variables (see Materials and methods), we performed receiver operating characteristic (ROC) analysis on models constructed using the five machine learning methods, based on their testing accuracy. The results showed that the performance of Scm^6^A based on the RF model achieved a median balanced accuracy of 0.91 across multiple tests on all m^6^A sites (Figure 1C), which was substantially higher than that of other classical machine learning methods. Moreover, we also conducted a comparative analysis between the RF model constructed solely using *trans*-acting regulators and that built solely using *cis*-regulatory elements. The median balanced accuracy of both models was found to be lower than that of the model created by integrating both types of effectors. The other four models achieved median balanced accuracies ranging from 0.8 to 0.9, suggesting that the regulatory network we constructed is reliable. Moreover, an example is shown in Figure 1D. The ROC curves of the five models demonstrated distinct prediction performances, with the RF model exhibiting the highest prediction efficiency. To further validate our findings, we conducted a randomized relabeling of samples and performed ROC analysis using the methodology described above, the area under the receiver operating characteristic curve (AUROC) and *R*^2^ values of the RF model significantly exceeded those generated by random permutations (Figure S1B and C), suggesting a significant level of accuracy that cannot be explained by random chance. Overall, we developed the best fitting single-cell m^6^A calculation method and named it Scm^6^A.

### Accuracy and reliability of Scm^6^A were further validated by m^6^A-seq in CD4^+^ and CD8^+^ T cells isolated from human PBMCs by MASC

**Figure 2:**
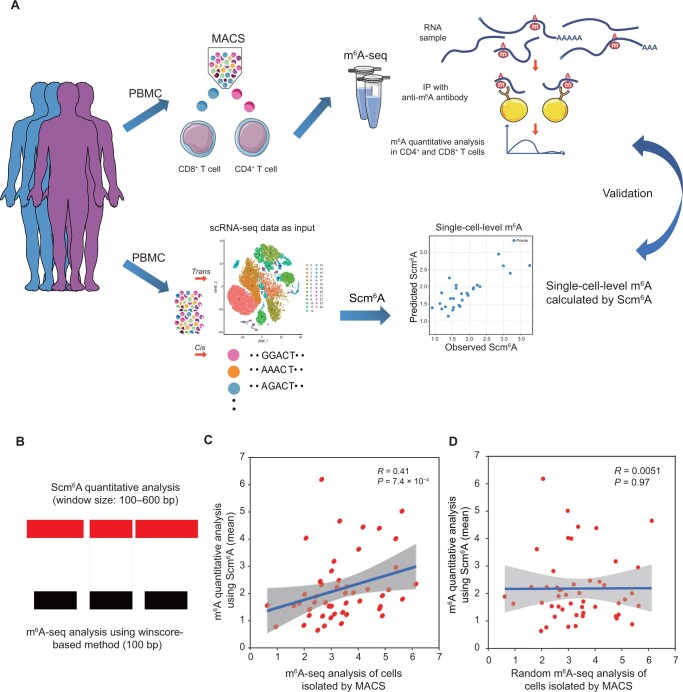
Verification of the reliability and accuracy of Scm^6^A **A**. Schematic diagram showing the framework of validation of Scm^6^A. **B**. Schematic diagram of the distribution of m^6^A peak lengths calculated by Scm^6^A and the winscore-based method using m^6^A-seq data. **C**. Correlation analysis between the Scm^6^A-calculated m^6^A levels and the winscore-based method-calculated m^6^A levels at the same m^6^A site. **D**. Correlation analysis between the Scm^6^A-calculated random m^6^A levels and the winscore-based method-calculated random m^6^A levels at the same m^6^A site. Scm^6^A, Single-cell m^6^A Analysis; PBMC, peripheral blood mononuclear cell; MACS, magnetic-activated cell sorting; scRNA-seq, single-cell RNA sequencing; m^6^A-seq, *N*^6^-methyladenosine sequencing; IP, immunoprecipitation.

We performed scRNA-seq analysis of PBMCs from four healthy participants, and extracted gene expression data for 593 reliable m^6^A regulators [18] as *trans*-acting input, as well as 42 m^6^A conserved sequences [18,26] as *cis*-acting input for Scm^6^A. Then, we calculated the single-cell-level m^6^A profiles in CD4^+^ and CD8^+^ T cells (**Figure 2**A). Simultaneously, we used a winscore-based m^6^A calculation method [18] to perform m^6^A-seq analysis of CD4^+^ and CD8^+^ T cells isolated by MACS from the same donors. To control technical biases in the regulatory network of *trans* m^6^A regulators to m^6^A sites in the m^6^A-seq libraries, including variations in sequencing lengths and RNA fragmentation lengths, we merged continuous Scm^6^A calculation peaks within the same gene, as described before [18]. Considering that different window sizes of two different calculation methods cannot be used for comparing the precise locations of m^6^A sites, we obtained 49 precisely matched m^6^A windows to compare the correlations of m^6^A levels within the same gene region (Figure 2B), and these m^6^A sites with same localizations on the transcriptome were the best choice for validating the accuracy and reliability of Scm^6^A. As we expected, there was a significant correlation between the m^6^A levels predicted by Scm^6^A and quantified by m^6^A-seq of cells isolated by MACS (Figure 2C). Moreover, there was no significant correlation between Scm^6^A and m^6^A-seq analysis results generated by random permutation of these m^6^A sites (Figure 2D), indicating a significant accuracy which cannot be explained by random chances.

To further validate the accuracy and reliability of Scm^6^A, we conducted a comparative analysis between Scm^6^A and the single-cell m^6^A sequencing (scm^6^A-seq) method recently published by Yang and colleagues [25]. Given that scm^6^A-seq employs mouse cleavage-stage embryo cells as its experimental subject, we initially converted mouse gene IDs to their corresponding human gene IDs and then subjected the gene expression data to standardized preprocessing, to enable an effective comparison between Scm^6^A and scm^6^A-seq analysis. Despite the difference in quantification methodologies, we harmonized the data through logarithmic transformations, ensuring that both Scm^6^A’s predictions and the scm^6^A-seq data provided in the study of Yang et al. [25] could be juxtaposed for analysis.

Further correlation analysis results indicated a significant positive relationship (*R* = 0.25, *P* = 1 × 10^−74^) between the predictions generated by Scm^6^A and the experimentally measured m^6^A levels in scm^6^A-seq. This finding not only validates the efficacy of the Scm^6^A model in capturing underlying patterns within the data to a certain extent but also strongly reinforces the reliability of Scm^6^A’s predictive outcomes. We subsequently shuffled the order of the m^6^A sites and then performed a correlation analysis between the computational results of Scm^6^A and scm^6^A-seq. The correlation in the shuffled matrix was nearly absent (*R* = 0, *P* = 0.58) (Figure S2A). In summary, Scm^6^A was proved to be a precise and dependable computational tool for single-cell m^6^A analysis. It’s cost-effectiveness, efficiency, and reliability make it a powerful tool with significant potential, offering researchers a rapid and effective means to explore the epigenetic features of cells.

### Identification of the potential role and landscape of m^6^A in CD4^+^ and CD8^+^ T cells through m^6^A-seq

It is widely accepted that the differentiation of T cell subtypes is associated with the expression of CD4 and CD8 [27]. Transcriptional regulation plays a critical role in regulating the fate choice of CD4^+^/CD8^+^ T cells [27]. Recently, some studies have reported that m^6^A has a broader impact on the dynamics of the RNA life cycle in T cell differentiation by regulating crucial genes involved in T cell differentiation [28].

**Figure 3:**
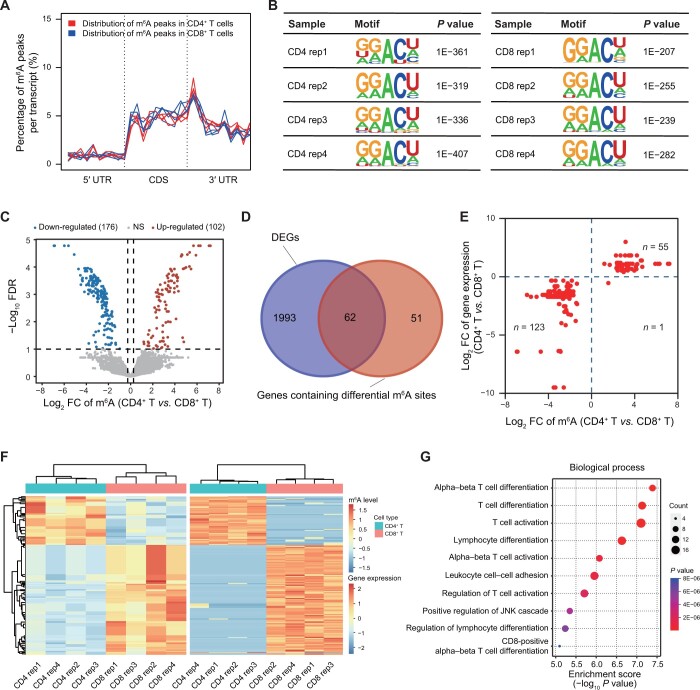
Bioinformatics analysis of m^6^A-seq results from MACS-isolated T cells in human PBMCs **A**. Normalized distributions of m^6^A peaks across the 5′ UTR, CDS, and 3′ UTR for CD4^+^ and CD8^+^ T cells from four samples. **B**. Representative motifs for CD4^+^ and CD8^+^ T cells in four samples. **C**. Volcano plot showing differential m^6^A sites (FC > 1.20 or < 0.83, FDR < 0.1). Dots in red and blue indicate up- and down-regulated m^6^A sites, respectively. **D**. Venn diagram showing the overlap between DEGs and genes containing differential m^6^A sites. **E**. Scatter plot showing the correlation between the expression levels of the 62 DEGs and the methylation levels of the corresponding 179 differential m^6^A sites. **F**. Heatmaps showing the expression of the 62 DEGs (right) and the methylation levels of the corresponding differential m^6^A sites (left). **G**. GO enrichment analysis of the 62 DEGs containing differential m^6^A sites. UTR, untranslated region; CDS, coding sequence; FDR, false discovery rate; FC, fold change; NS, not significant; DEG, differentially expressed gene; GO, Gene Ontology.

Typically, a combination analysis of m^6^A-seq and RNA sequencing (RNA-seq) is used to identify the potential role and mechanism of m^6^A-regulated genes in biological processes. To further validate the reliability of Scm^6^A, we performed m^6^A feature analysis using m^6^A-seq data from cells isolated by MACS and RNA-seq analysis to identify the potential differences in the role and landscape of m^6^A in CD4^+^ and CD8^+^ T cells. As shown in **Figure 3**A, the m^6^A peaks of CD4^+^ T cells tended to be enriched near stop codons, while the m^6^A peaks of CD8^+^ T cells were enriched in coding regions and start codons, suggesting that the different T cell types may have different m^6^A regulators controlling the m^6^A-mediated gene expression. Motif enrichment analysis of CD4^+^ and CD8^+^ T cells showed that m^6^A peaks in CD4^+^ T cells more tended to be enriched in the GGACU motif than those in CD8^+^ T cells. To be more specific, the *P* values for motif enrichment analysis in CD4^+^ T cells ranged from 1E−407 to 1E−319, while the *P* values in CD8^+^ T cells ranged from 1E−282 to 1E−207 (Figure 3B). Then, we performed differential m^6^A analysis and differential expression analysis using m^6^A-seq and input data [18]. We identified 2055 differentially expressed genes (DEGs) and 113 genes containing 278 differentially methylated m^6^A sites, with 62 genes overlapped (Figure 3C and D). We found that the expression levels of these 62 genes were positively related to the methylation levels of the corresponding 179 differential m^6^A sites (Figure 3E and F). As expected, these potential m^6^A-regulated genes were enriched in pathways related to T cell differentiation and cell differentiation, among others (Figure 3G), suggesting that m^6^A controls the expression of T cell differentiation-related genes.

### Identification of the potential role and landscape of m^6^A in CD4^+^ and CD8^+^ T cells through Scm^6^A

**Figure 4:**
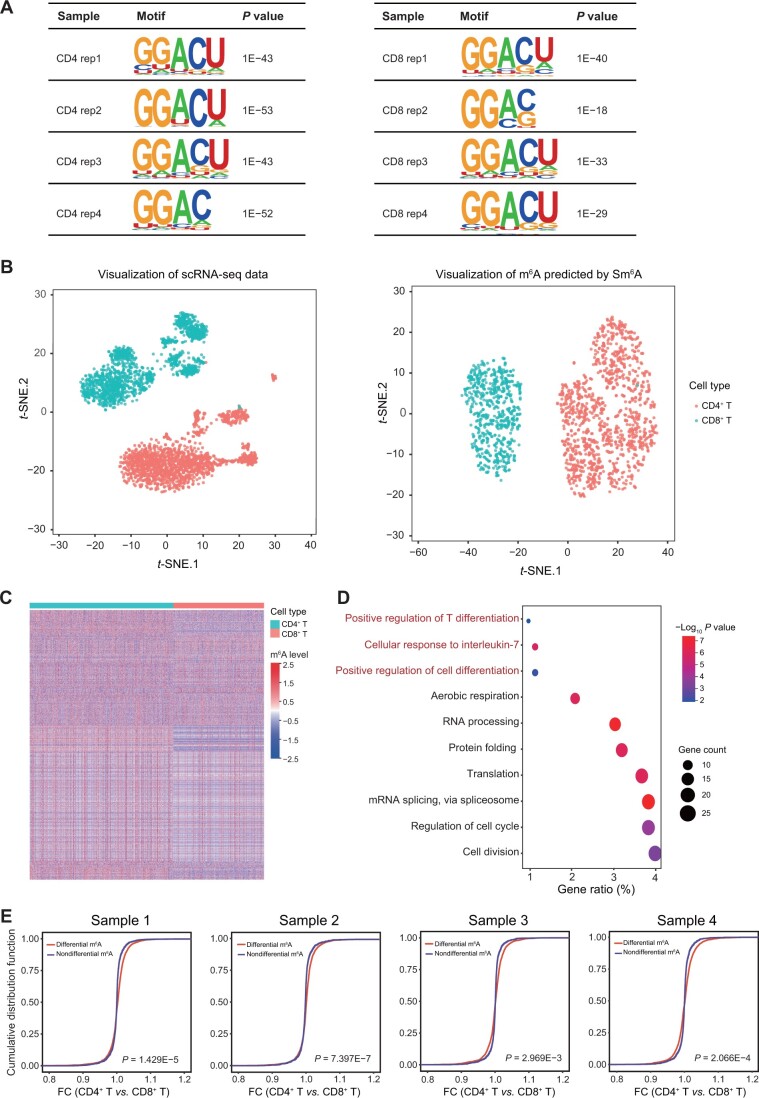
Bioinformatics analysis of Scm^6^A results from scRNA-seq data of T cells in human PBMCs **A**. Representative motifs for CD4^+^ and CD8^+^ T cells in four samples. **B**. *t*-SNE maps of scRNA-seq data (left) and m^6^A predicted by Scm^6^A (right). **C**. Cluster heatmap showing the differential m^6^A sites (CD4^+^ T *vs.* CD8^+^ T: FC > 1.20 or < 0.83, FDR < 0.1) predicted by Scm^6^A. **D**. GO enrichment analysis of the DEGs containing the differential m^6^A sites predicted by Scm^6^A. **E**. Cumulative distribution function plots of differential and nondifferential m^6^A sites. *t*-SNE, *t*-distributed stochastic neighbor embedding.

Next, we performed a combination analysis of scRNA-seq and Scm^6^A to identify the potential role and landscape of m^6^A in CD4^+^ and CD8^+^ T cells. As shown in **Figure 4**A, motif enrichment analysis revealed that m^6^A peaks calculated by Scm^6^A exhibited a tendency to be more highly enriched in the GGACU motif in CD4^+^ T cells than in CD8^+^ T cells, consistent with the enrichment pattern of m^6^A peaks identified through m^6^A-seq analysis (Figure 3B). To comprehensively analyze the m^6^A landscape at single-cell resolution, we performed unsupervised clustering analysis of single-cell-level m^6^A in CD4^+^ and CD8^+^ T cells identified by scRNA-seq combined with Scm^6^A. We observed two clusters of single-cell m^6^A profiles, which were clearly separated according to the cell types (Figure 4B). Moreover, we predicted the m^6^A levels of CD4^+^ and CD8^+^ T cells from a single sample at single-cell resolution and used cluster heatmaps to visualize the within-group similarity of the same cell type and the heterogeneity between groups of different cell types (Figure 4C). The DEGs containing the differential m^6^A modifications were enriched in T cell differentiation and cell differentiation (Figure 4D). We also found that the expression levels of the differential m^6^A-deposited DEGs were positively related to the methylation levels of the differential m^6^A sites (Figure 4E), consistent with the results obtained from m^6^A-seq analysis using MACS-isolated cells (Figure 3E and F). These findings further underscore the reliability of Scm^6^A as a method for single-cell-level m^6^A analysis.

### Application of Scm^6^A in different lung cancer subtypes revealed the potential role and regulators of m^6^A in exhausted CD8^+^ T cells

The differentiation of exhausted CD8^+^ T cells leads to attenuated effector function of cytotoxic CD8^+^ T cells, resulting in their inability to control tumor progression during the advanced stage [29]. Furthermore, it has been reported that m^6^A plays a crucial role in regulating T cell homeostasis [8]. However, our current understanding of the different m^6^A profiles in distinct subtypes of T cells and its role in exhausted CD8^+^ T cells is currently limited [8].

**Figure 5:**
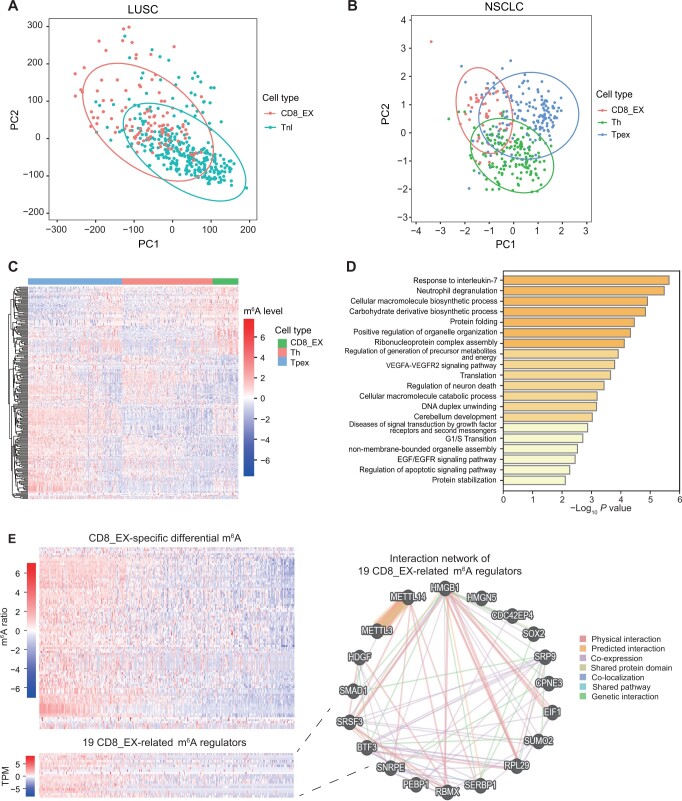
Exploration of the m^6^A profiles of different lung cancer subtypes at single-cell level using scRNA-seq combined with Scm^6^A **A**. and **B**. PCA of the m^6^A profiles predicted by Scm^6^A at single-cell level for LUSC (A) and NSCLC (B). Each color represents a cell. The ellipse around the group represents the confidence region. **C**. Heatmap showing differential m^6^A sites between distinct T cell subtypes in NSCLC. **D**. GO enrichment analysis of genes containing the CD8_EX-specific differential m^6^A sites. **E**. Upper: heatmap showing the m^6^A ratios of the m^6^A peaks within the genes containing the CD8_EX-specific differential m^6^A sites. Lower: heatmap showing the gene expression levels of the 19 CD8_EX-related m^6^A regulators that were significantly correlated with the methylation levels of the CD8_EX-specific differential m^6^A sites. Right: GeneMANIA interaction network of 19 CD8_EX-related m^6^A regulators with physical interaction, predicted interaction, co-expression, shared protein domain, co-localization, genetic interaction, and shared pathway. Dim gray nodes represent query regulators. PCA, principal component analysis; PC, principal component; LUSC, lung squamous carcinoma; NSCLC, non-small cell lung cancer; CD8_EX, exhausted CD8^+^ T cell; Tnl, naïve-like T cell; Th, helper T cell; Tpex, progenitor exhausted CD8^+^ T cell; TPM, transcripts per million.

Herein, we employed Scm^6^A to further explore the m^6^A landscapes of exhausted CD8^+^ T cells and other cell types across different lung cancer subtypes, including lung squamous carcinoma (LUSC) (**Figure 5**A) and non-small cell lung cancer (NSCLC) (Figure 5B). Our findings revealed differences in the m^6^A profiles and molecular features of exhausted CD8^+^ T cells compared to other T cell subtypes in both LUSC and NSCLC (Figure 5A–C, Figure S2B). Interestingly, genes containing the exhausted CD8^+^ T cell (CD8_EX)-specific differential m^6^A sites were enriched in IL-7 pathway, which is associated T cell homeostasis (Figure 5D). By investigating the regulatory network that we constructed (Figure 1A), it became evident that these CD8_EX-specific differential m^6^A sites are associated with 19 m^6^A regulators (hereafter referred to as CD8_EX-related m^6^A regulators), including METTL3, METTL14, and HMGB1 (Figure 5E). Moreover, we observed a significant positive correlation between the expression levels of these 19 regulators and the methylation levels of the CD8_EX-specific differential m^6^A sites (Figure 5E). Therefore, we concluded that CD8_EX-related m^6^A regulator-mediated m^6^A modifications may regulate T cell homeostasis through targeting IL-7. Consistent with this result, Li et al. also found that METTL3-mediated m^6^A modifications controlled T cell homeostasis and differentiation by targeting IL-7, proving the reliability of our analysis results [8]. Notably, HMGB1, acting as a pivotal node in CD8_EX-related m^6^A regulation (Figure 5E), has previously been reported to influence the infiltration of CD8^+^ T cells in NSCLC [30], further supporting the reliability of our conclusions.

The dysregulation of immune responses in COVID-19 patients has emerged as a primary factor affecting symptoms and mortality rates. Thus, investigating relevant immune cells has become a focal point in combatting this disease. In this context, we applied Scm^6^A to a COVID-19 dataset from the study by Yuan and his colleagues [24]. From this dataset, we randomly selected a COVID-19-positive patient sample that had undergone fluorescence activated cell sorter (FACS) selection to isolate CD3^+^ T cells and CD19^+^ B cells from fresh PBMCs. Using the Seurat package [31], we imported and standardized the single-cell data, and then input the standardized data into Scm^6^A for prediction. The resulting Uniform Manifold Approximation and Projection (UMAP) plot of m^6^A predictions clearly delineated the classification of T cells and B cells (Figure S3A), highlighting a marked divergence in m^6^A landscapes between these cell types. Subsequent Gene Ontology (GO) functional enrichment analysis of the genes containing the differential m^6^A sites revealed significant enrichment in terms of “SARS-CoV infections” and “Viral infection pathways”, aligning with expectations and confirming the accuracy of Scm^6^A’s predictions (Figure S3B). These findings contribute to the dissection of the immune response in COVID-19 patients at a single-cell m^6^A resolution, enabling a deeper exploration of the pathogenic mechanisms at play.

These results provide a fresh perspective on the comprehensive profiles of m^6^A and corresponding regulators in exhausted CD8^+^ T cells and other T cell subtypes. Obviously, Scm^6^A has broaden applications in the identification of subtypes of different cell types, and it may redefine some novel cell types and further reveal the potential role of m^6^A in cellular differentiation. This opens up exciting possibilities for future research in this field.

## Discussion

It has been reported that there is high heterogeneity in the abundance of m^6^A across individual cells [32]. However, a reliable and convenient method for detecting m^6^A at the single-cell level is currently lacking. In this study, we developed Scm^6^A, a single-cell m^6^A detection method based on the *trans* and *cis* information that we previously constructed. We validated the accuracy of the Scm^6^A method using m^6^A-seq data from MACS-isolated cell types. Subsequently, we applied Scm^6^A to scRNA-seq data from lung cancer. Our analysis reveals that m^6^A is highly variable across different subtypes of T cells in lung cancer tissues. It also provides new ideas for the development of new single-cell calculation methods for other RNA methylation modifications, such as 5-methylcytosine (m^5^C), 7-methylguanosine (m^7^G), and *N*^1^-methyladenosine (m^1^A). Our attempts on the COVID-19 dataset further demonstrate that Scm^6^A can effectively predict m^6^A levels and, in turn, distinguish between B cells and T cells. Moreover, we will further improve the application scope of Scm^6^A to analyze the single-cell-level m^6^A in other species, including monkeys and plant species.

Recently, Matthew et al. developed a method named scDART-seq to identify transcriptome-wide YTH-binding m^6^A sites in single cells by inducing APOBEC1-YTH expression [32]. However, this method can only be used to detect YTH-binding m^6^A sites, not all the m^6^A sites. In addition, this method requires the expression of APOBEC1-YTH in targeted cells, which is not compatible with single-cell sequencing data. Moreover, scDART-seq can detect some false-positive m^6^A sites [32]. Due to the limitations of scDART-seq, there are no convenient and swift methods for identifying transcriptome-wide m^6^A sites and levels in individual cells with a low false discovery rate (FDR) [33]. Comparing with scDART-seq, Scm^6^A offers a more accurate and convenient approach for quantifying m^6^A at the single-cell level. Therefore, Scm^6^A has more potential to be widely used in m^6^A-related research. Combining analysis of scRNA-seq with Scm^6^A also provides the pathway for researchers to investigate the detailed m^6^A regulation mechanisms at the single-cell level. Moreover, Scm^6^A displays high true positive rate with AUROC = 0.91, suggesting that Scm^6^A can detect few false-positive m^6^A sites.

The role of m^6^A in T cell differentiation and T cell homeostasis has attracted much attention. As a key step of cancer immune evasion, there is a lack of research on the epigenetic mechanisms related to T cell exhaustion [34], *e.g.*, whether m^6^A is involved in regulating T cell exhaustion. By using Scm^6^A, we found that the m^6^A profiles were distinct among the exhausted T cells and other subtypes of T cells, indicating that m^6^A plays an essential role in the progression of T cell exhaustion. The impact of m^6^A on T cell differentiation and activation deserves further discussion using our Scm^6^A method. Considering the discovery of pharmacological inhibition of METTL3 [35], it is plausible that we may combat T cell exhaustion by m^6^A-dependent gene regulation in the future. In fact, our method not only provides a single-cell m^6^A method for classifying T cells but also extends to other cell type subpopulations, including B cells, dendritic cells, and macrophages. We believe that Scm^6^A will be used to study the role of single-cell m^6^A in a variety of diseases and cell types.

Scm^6^A is based on a m^6^A-seq-trained machine learning method and is antibody free. Even Scm^6^A is not at single-base resolution for every single transcript yet, we will incorporate new features of m^6^A identification using nanopore sequencing and miCLIP-m^6^A to Scm^6^A in the future [36–40]. We believe that it will make Scm^6^A a more powerful tool at single-base resolution for every single transcript. Furthermore, we will try to collect more m^6^A-seq data for training to improve the accuracy of Scm^6^A.

In summary, this study provides a novel approach for calculating RNA methylation at the single-cell level. Combined with other single-cell multiomics techniques, Scm^6^A will open up a new way for m^6^A research at the single-cell level which will be expected to significantly contribute to our understanding of the role of single-cell m^6^A in various biological processes.

## Materials and methods

### Data preprocessing and machine learning algorithms

In accordance with our previous research, we collated and analyzed raw sequencing data from 1 × 10^4^ m^6^A-seq libraries [immunoprecipitation (IP) and input] obtained from 25 unique cell lines, which were downloaded from the Genome Sequence Archive (GSA, https://ngdc.cncb.ac.cn/gsa/). We identified comprehensive *trans* regulators in the m^6^A regulatory network and *cis* sequence features of the m^6^A sites using these m^6^A-seq data [18]. Herein, we developed a single-cell-level calculation method based on machine learning models using this network from the expression levels of the m^6^A regulators to the m^6^A level. As *cis*-acting regulatory sequences, position probability matrices of each m^6^A site were used as input to the model [41]. To solve the frequent NA values in m^6^A data, we first counted the percentage of missing values in each row and column and then deleted rows or columns with missing values greater than 10%. Following the data filtering process, the expression matrix consisting of 4162 m^6^A sites was obtained.

For the development of machine learning models, we established a one-to-one correspondence between the expression of m^6^A regulators and the m^6^A matrices to construct models. Meanwhile, considering the regulation of *cis*-acting elements to m^6^A, we converted the motif sequence features of each m^6^A site into a matrix as part of the training data of the algorithm. In this study, five machine learning algorithms, including RF, LR, KNN, LinearSVR, and SVR_poly, were used to determine the most effective method. The scikit-learn toolkit (v1.0.2) was used to train these machine learning models. Out of the overall dataset, 70% was randomly allocated to serve as the training set for model development, while the remaining 30% was reserved for the test set to validate the model’s performance. The best parameters were selected through grid search and five-fold cross validation.

### m^6^A-seq data processing

We established m^6^A-seq libraries (IP and input) of CD4^+^ and CD8^+^ T cells isolated from the same individuals using MACS, and obtained four different sets of samples. The reads generated by m^6^A-seq were mapped to the hg38 human reference genome using HISTA2 with default values for parameters (v2.1.0) [42]. We calculated the transcripts per million (TPM) of input libraries of Ensembl annotated genes using StringTie and then performed quantile normalization across all samples [43].

To accurately identify the m^6^A sites in CD4^+^ and CD8^+^ T cells, we improved the winscore method previously published by Dominissini and his colleagues [33]. We determined the sliding window with a window fraction (enrichment fraction) > 2 in the sample as the m^6^A peak. Since low expression windows may be accompanied by technical problems with unreliable winscore, we adjusted the windows with low reads per kilobase of transcript per million mapped reads (RPKMs) by adding 1 to the RPKM of each window in both IP and input libraries before winscore calculation. After identifying the m^6^A peaks across the samples, we merged consecutive m^6^A peaks within the same gene, and then divided the merged peaks with more than 5 consecutive sliding windows (300 bp) into multiple peaks, spanning no more than 5 sliding windows to eliminate the problem of possible false positives. After the abovementioned analyses, we finally obtained the m^6^A matrices of m^6^A levels in CD4^+^ and CD8^+^ T cells, respectively.

### scRNA-seq data processing

The “Seurat” package [31] was used to analyze the scRNA-seq data of CD4^+^ and CD8^+^ T cells. Genes expressed in fewer than 3 cells and cells expressing fewer than 200 genes were excluded. Since cells with a high proportion of mitochondria-derived genes, a low number of detected genes, and a high proportion of unmapped or multi-mapped reads are often damaged or dying cells, which will affect the subsequent scRNA-seq analysis, we performed quality control (QC) on the data to filter out the unqualified data. The parameters used were as follows: nFeature_RNA > 200 or < 4000, nCount_RNA > 200 or < 20,000, percent.mt < 25. To correct the variability of meaningful reads obtained by scRNA-seq across different cells, we normalized the expression using the “NormalizeData” function and calculated the top 1500 highly expressed variant genes by the “FindVariableFeatures” function.

To visualize and interpret the high-dimensional gene expression data, principal component analysis (PCA) and *t*-distributed stochastic neighbor embedding (*t-*SNE) were used for visualizing scRNA-seq data of CD4^+^ and CD8^+^ T cells. A total of 24 cell clusters were obtained after visualization. Using Cell Ranger (https://support.10xgenomics.com/single-cell-dna/software/overview/welcome), we defined the cluster of cells with high CD4 expression as CD4^+^ T cells and the cluster of cells with high CD8A and/or CD8B expression as CD8^+^ T cells. After counting the marker gene expression levels of cells from the four samples (Sample 1–Sample 4) and sorting the cells, we defined cluster 1 in the 24 clusters as CD8^+^ T cells, and clusters 6, 9, 10, 11, 16, 18, 21, and 22 as CD4^+^ T cells according to their marker gene expression levels. To obtain DEGs, we used “DESeq2” was used to perform differential gene expression analysis between CD4^+^ and CD8^+^ T cells obtained by scRNA-seq, and the parameters used were as follows: FDR < 0.1, fold change (FC) > 1.20 or < 0.83 (CD4^+^ T *vs.* CD8^+^ T). After that, we used the “limma” package to analyze the genes containing the differential m^6^A sites between CD4^+^ and CD8^+^ T cells obtained by m^6^A-seq, and the parameters used were as follows: FDR < 0.1, FC > 1.20 or < 0.83 (CD4^+^ T *vs.* CD8^+^ T). We downloaded lung cancer scRNA-seq data from the Gene Expression Omnibus (GEO: GSE148071) [44] as external validation data, which contained 42 scRNA-seq datasets from tissues of stage III/IV NSCLC patients. We performed lung cancer-related single-cell m^6^A analysis based on these scRNA-seq data. Through Scm^6^A, we calculated single-cell m^6^A levels using the expression levels of m^6^A regulators and position probability matrices of m^6^A sites as input from these scRNA-seq data.

### Human CD4^+^/CD8^+^ T cell sorting

Human CD4^+^/CD8^+^ T cells were obtained from PBMCs of adult donors in good health. The PBMCs were first isolated from whole blood by density gradient centrifugation (ficoll 1.077 g/ml; Ficoll-Paque PLUS, Sigma-Aldrich, St. Louis, MO) and then maintained in RPMI 1640 medium (Catalog No. R8438, Solarbio, Beijing, China) supplemented with 15% fetal bovine serum (FBS; Catalog No. F4135, Sigma-Aldrich) and 1% penicillin/streptomycin (Catalog No. P1400, Solarbio). Subsequently, CD4^+^/CD8^+^ T cells were isolated from PBMCs using MACS beads (Catalog No. 130-091-101, Miltenyi Biotec, Bergisch Gladbach, Germany). Briefly, PBMCs were bound to CD4 microbeads (20 µl of microbeads/1 × 10^7^ cells) for 15 min at 4°C. After washing with washing buffer, the cells were resuspended in 500 µl of washing buffer, passed through an LS column (Catalog No. 130-042-401, Miltenyi Biotec) attached to a magnetic stand (Catalog No. 130-042-201, Miltenyi Biotec), and were washed three times. To elute targeted cells, the column was washed with buffer after being removed from the magnetic field. The approach was validated by MACS. The sorted targeted cells were used for single-cell polymerase chain reaction (PCR) analysis and sequencing.

### GO enrichment analysis

To further investigate the mechanisms associated with the differences observed in different T cells in healthy humans, we performed GO functional enrichment analysis in Database for Annotation, Visualization and Integrated Discovery (DAVID, https://david.ncifcrf.gov/) by using the genes containing the differential m^6^A sites, and took the top 10 items of the biological process ranked in ascending order of FDR as the results [45].

### Motif enrichment and distribution analyses of m^6^A peaks

Hypergeometric Optimization of Motif Enrichment (HOMER, http://homer.ucsd.edu/homer/) software was used for motif enrichment analysis, using the Browser Extensible Data (BED) format file obtained earlier as the input file. The distribution of m^6^A peaks was plotted on a mega gene with 10 bins in the 5′ untranslated region (5′ UTR), coding sequence (CDS), and 3′ UTR, using the methods described in our previous study [18].

### Statistical analysis

Statistical analysis was performed using R (v4.0.2, https://www.r-project.org/). The ROC curves were plotted and the AUROC values were calculated using the pROC package to compare the efficacy of each model. The AUROC values were calculated by randomly sampling the true and predicted values. Values were labeled as 1 if the difference between the predicted m^6^A value and the true value was no more than 0.5, and labeled as 0 if the difference fell outside the range. The evaluation indicators of the five machine learning regressors did not conform to the normal distribution, so the quartile was used for statistics.

## Ethical statement

The studies involving human participants were reviewed and approved by the Ethics and Human Subjects Committee of Guangxi Medical University, China (Approval No. 20210092). The patients/participants provided their written informed consent to participate in this study.

## Code availability

The code associated with this research is available on GitHub ( https://github.com/Ansanqi/Scm6A).

## Supplementary Material

qzae039_Supplementary_Data

## Data Availability

The raw data of the m^6^A-seq and scRNA-seq data have been deposited in the Genome Sequence Archive for Human [46] at the National Genomics Data Center, Beijing Institute of Genomics, Chinese Academy of Sciences / China National Center for Bioinformation (GSA-Human: HRA006292), and are publicly accessible at https://ngdc.cncb.ac.cn/gsa-human/.

## CRediT author statement

**Yueqi Li:** Formal analysis, Visualization, Writing – original draft, Writing – review & editing. **Jingyi Li:** Formal analysis, Writing – original draft. **Wenxing Li:** Formal analysis, Writing – original draft. **Shuaiyi Liang:** Formal analysis, Writing – original draft. **Wudi Wei:** Formal analysis, Writing – original draft. **Jiemei Chu:** Formal analysis. **Jingzhen Lai:** Formal analysis. **Yao Lin:** Visualization. **Hubin Chen:** Visualization. **Jinming Su:** Visualization. **Xiaopeng Hu:** Resources. **Gang Wang:** Resources. **Jun Meng:** Resources. **Junjun Jiang:** Resources, Conceptualization. **Li Ye:** Resources, Conceptualization. **Sanqi An:** Writing – original draft, Writing – review & editing, Conceptualization, Funding acquisition. All authors have read and approved the final manuscript.

## ORCID

0000-0002-2640-4906 (Yueqi Li)

0009-0002-6440-5106 (Jingyi Li)

0000-0001-9984-8439 (Wenxing Li)

0009-0007-6343-9933 (Shuaiyi Liang)

0000-0001-6703-4529 (Wudi Wei)

0000-0003-2942-0490 (Jiemei Chu)

0000-0003-2054-6396 (Jingzhen Lai)

0000-0003-1728-3791 (Yao Lin)

0009-0003-0216-7138 (Hubin Chen)

0000-0003-0178-2115 (Jinming Su)

0000-0002-6533-8024 (Xiaopeng Hu)

0000-0001-7264-2457 (Gang Wang)

0009-0008-8890-1220 (Jun Meng)

0000-0003-4905-9348 (Junjun Jiang)

0000-0001-7688-4867 (Li Ye)

0000-0002-3177-213X (Sanqi An)
