# Supplementary material for: Scm6A: A Fast and Low-cost Method for Quantifying m6A Modifications at the Single-cell Level
Source: Genomics Proteomics Bioinformatics. 2024 Jun 7;22(4):qzae039. doi: 10.1093/gpbjnl/qzae039 (PMC12016562; doi:10.1093/gpbjnl/qzae039)
Supplement: qzae039_Supplementary_Data [file qzae039_supplementary_data.zip › Supplementary material captions.docx]

**Supplementary material**

**Figure S1 A comparative analysis of m^6^A calculation methods based on the RF model and other models**

**A.** Dot plots show the correlations between predicted m^6^A level and true m^6^A level for different models. **B.** and **C.** Boxplots show R-squared (B) and AUROC (C) of RF model using corresponding m^6^A regulators or random permutation to calculate single cell m^6^A.

**Figure S2 Application of Scm^6^A in datasets pertaining to mouse cleavage-stage embryos and lung cancer**

**A.** Scatter plot of correlation between Scm^6^A prediction results and the scm^6^A-seq sequencing outcomes (left), and random correlation scatter plot of both (right). **B.** Representative motifs for CD8_EX, Th, and Tpex cells in NSCLC samples. CD8_EX, exhausted CD8^+^ T cell; Th, T helper cell; Tpex, progenitor exhausted CD8^+^ T cell.

**Figure S3 Application of Scm^6^A in** **COVID-19 datasets**

**A.** UMAP plot of m^6^A predicted by Scm^6^A. **B.** GO enrichment analysis of genes with differential m^6^A modifications. COVID-19, the coronavirus disease 2019; UMAP, Uniform Manifold Approximation and Projection.

**Table S1 The RBP information**
