# Supplementary figures and images for: Scm6A: A Fast and Low-cost Method for Quantifying m6A Modifications at the Single-cell Level
Source: Genomics Proteomics Bioinformatics. 2024 Jun 7;22(4):qzae039. doi: 10.1093/gpbjnl/qzae039 (PMC12016562; doi:10.1093/gpbjnl/qzae039)

A

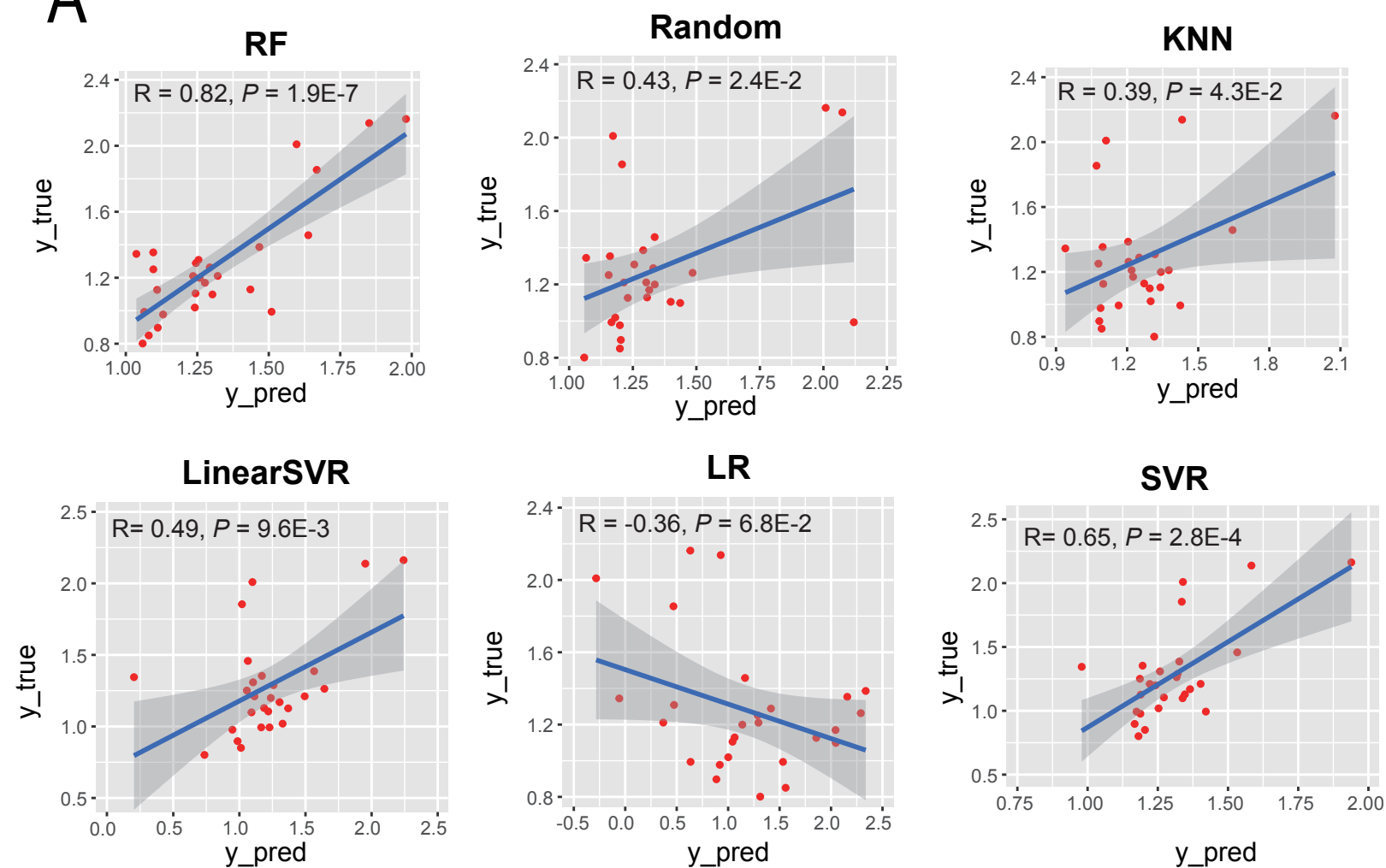

B

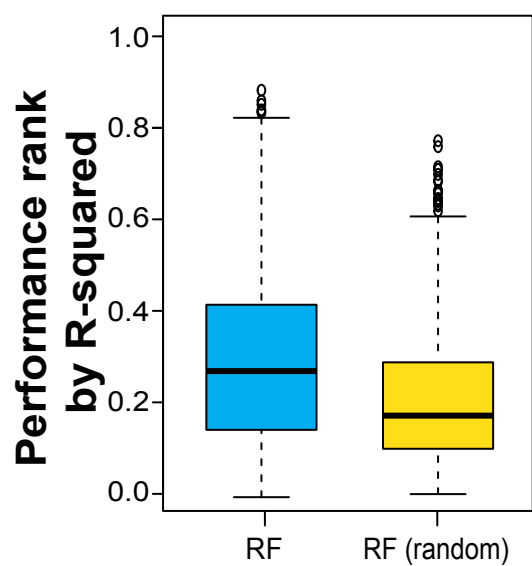

C

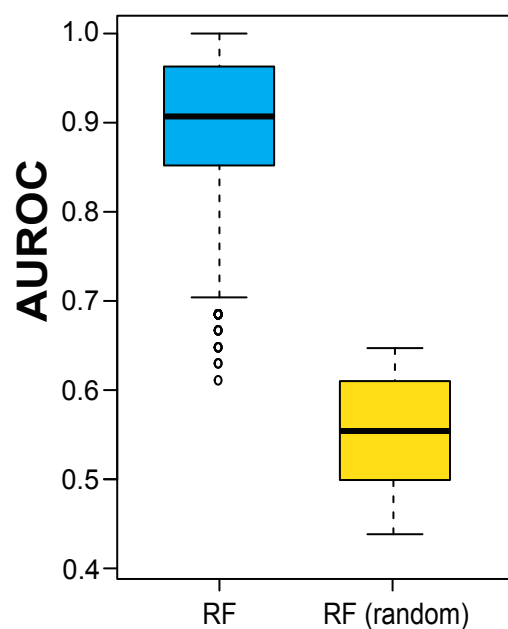

Supplement: qzae039_Supplementary_Data [file qzae039_supplementary_data.zip › Figure S1.pdf]

A

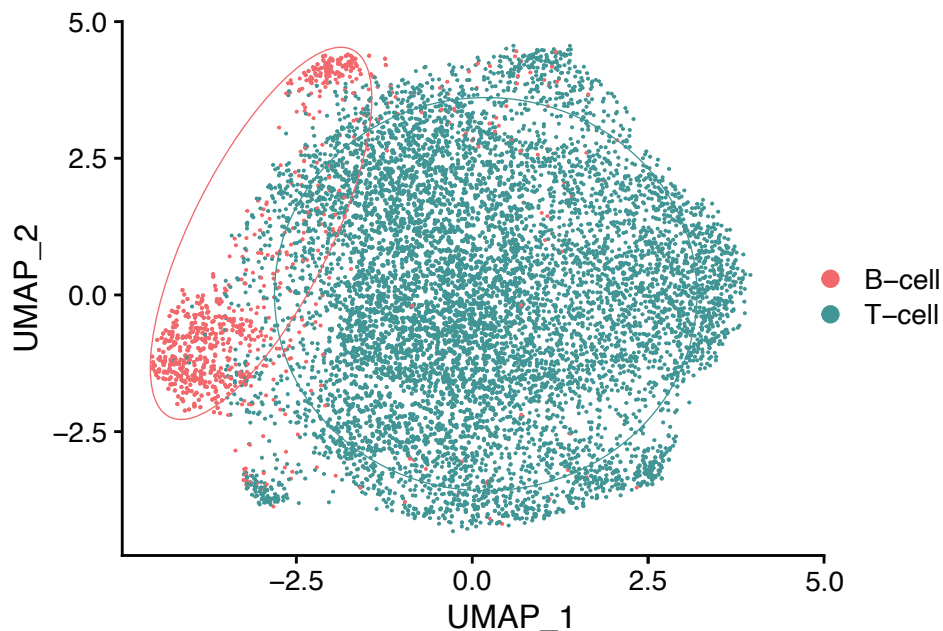

B

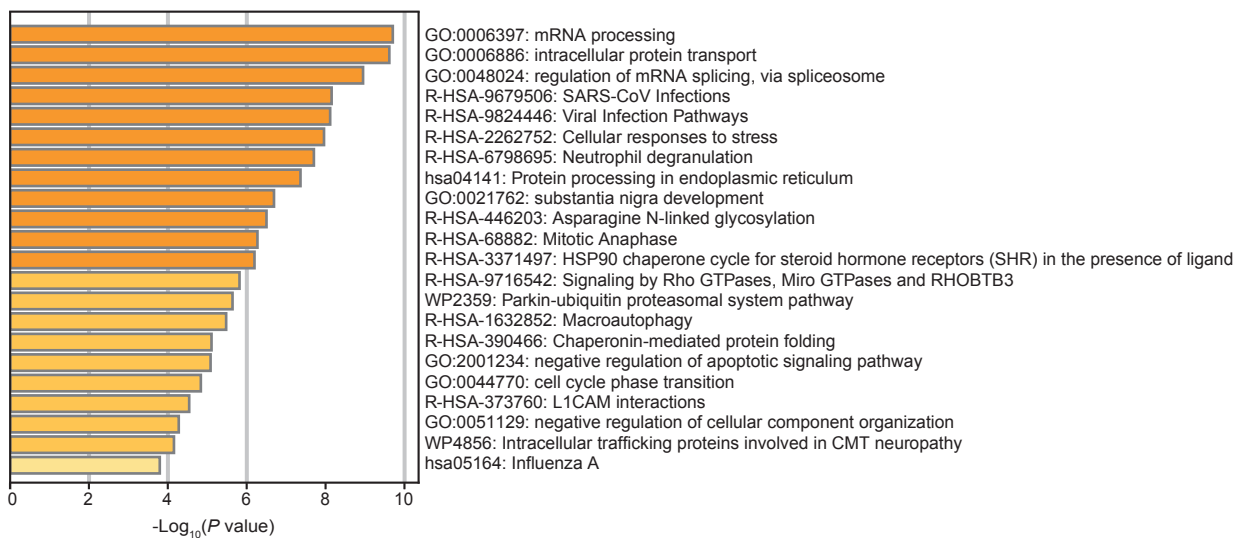

Supplement: qzae039_Supplementary_Data [file qzae039_supplementary_data.zip › Figure S3.pdf]

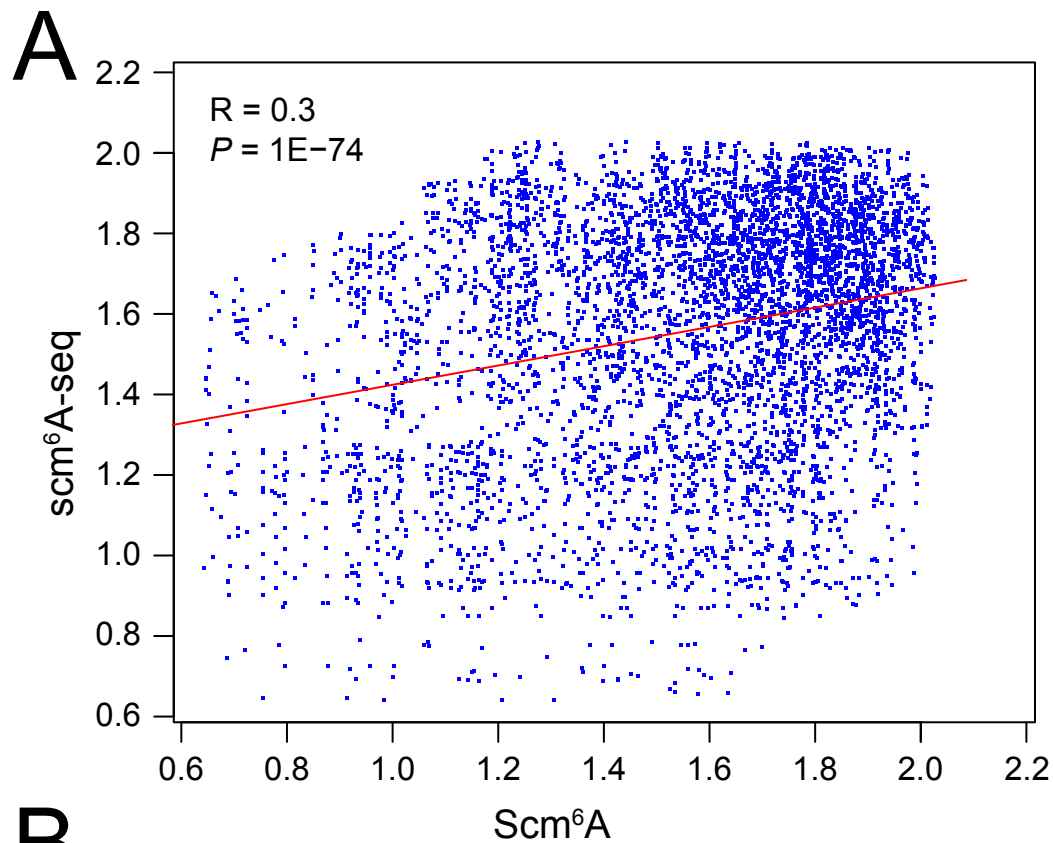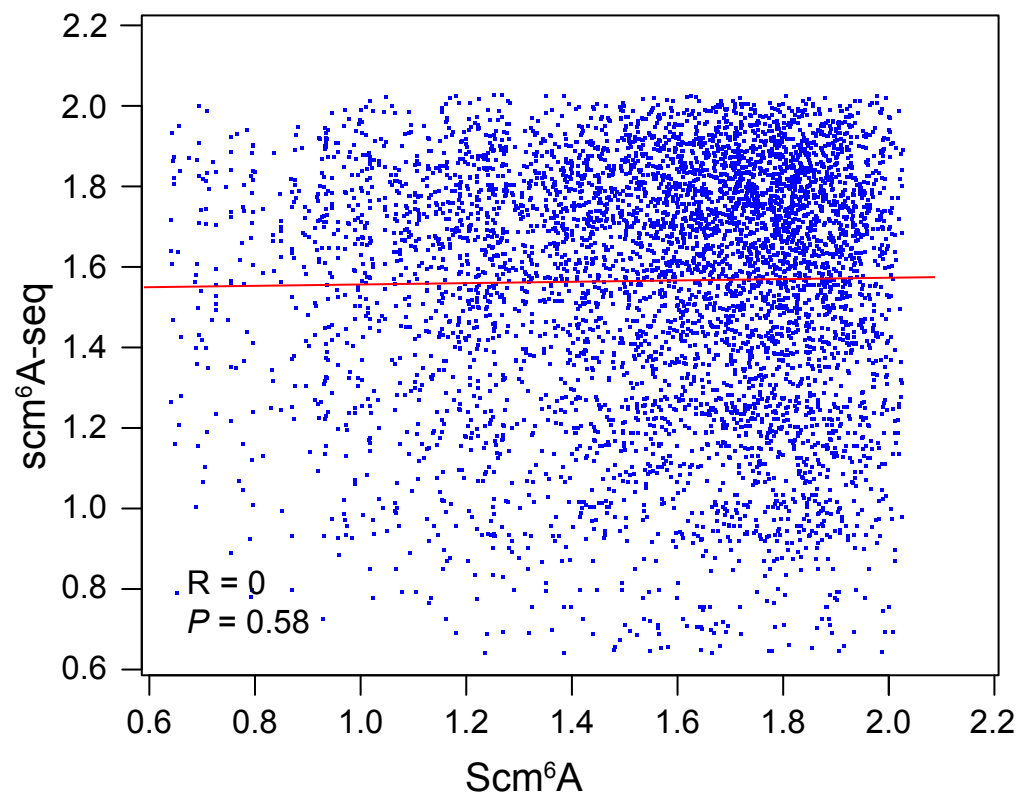

**B**

| Cluster | Motif | <i>P</i> value |
|---------|-------|----------------|
| CD8_EX  |       | 1E-3           |
| Th      |       | 1E-7           |
| Tpex    |       | 1E-10          |

Supplement: qzae039_Supplementary_Data [file qzae039_supplementary_data.zip › Figure S2.pdf]
